# Management challenges with brown tumor of primary hyperparathyroidism masked by severe vitamin D deficiency: a case report

**DOI:** 10.1186/s13256-016-0933-4

**Published:** 2016-06-09

**Authors:** Marya Hussain, Montasir Hammam

**Affiliations:** 1 King Fahad Specialist Hospital, Dammam, Saudi Arabia; 2 Department of Internal Medicine, King Fahad Specialist Hospital, Dammam, Saudi Arabia

**Keywords:** Hyperparathyroidism, Hypercalcemia, Vitamin D, Brown tumor

## Abstract

**Background:**

Hyperparathyroidism is a disease characterized by excessive secretion of parathyroid hormone, the hormone responsible for calcium and phosphate homeostasis in the body. It can be of three types: primary, secondary, or tertiary. It is essential to bear in mind that in any one patient more than one type of hyperparathyroidism may be found, which may create perplexity regarding the etiology of the case. Hyperparathyroidism can become apparent early in its course when a patient presents with symptoms of abdominal pain, recurrent renal calculi, repeated fractures, or behavior changes. It is generally accepted that bone involvement is a late manifestation of primary hyperparathyroidism. It is imperative to consider that some patients, such as our patient described in this report, may be previously asymptomatic clinically and on the basis of laboratory findings and present with only late skeletal manifestations. Brown tumors are one of the mimickers of lytic lesions of the jaw and need to be ruled out early in the course of management. Researchers in several studies published in high-impact journals have recommended the use of high-dose vitamin D as safe in patients with primary hyperparathyroidism without the risk of raising calcium levels significantly. In our patient, we observed considerable hypercalcemia after high-dose vitamin D therapy, and we propose exercising discretion with the use of high-dose therapies.

**Case presentation:**

We report a case of a 21-year-old Arab woman with a brown tumor who presented with hypocalcaemia. She presented with a mixed picture of primary hyperparathyroidism and severe vitamin D deficiency.

**Conclusions:**

Brown tumors, although thought to be a forgotten entity with the advent of early screening for hypercalcemia, is still prevalent, as a handful of patients may present late in the disease course with no early markers, such as in our patient. We emphasize using a holistic approach for early diagnosis and adopting a restricted attitude to treating these benign entities, especially in the context of cosmesis for sensitive locations such as the face. In addition, we express caution in using daily supplementation with a high vitamin D dose to improve vitamin D status and decrease parathyroid hormone.

## Background

Primary hyperparathyroidism (PHPT) is one of the most common endocrine disorders encountered in endocrinology practice today. The frequencies of various parathyroid lesions underlying the hyperfunction are adenoma (80–95 %), primary hyperplasia (5–10 %), and parathyroid carcinoma (1 %) [1]. PHPT is a disease of adulthood seen mostly in those older than 50 years of age, and it is more common in women [2]. Eighty percent of patients are identified on the basis of an incidental discovery of hypercalcemia. In severe and late stages of hyperparathyroidism, skeletal changes can be observed. These changes include an increased number of osteoclasts, which erode bone matrix and increase bone resorption. This in turn causes increased osteoblastic activity, which results in new bone formation. The bone loss predisposes individuals to microfractures and hemorrhages, which result in an influx of macrophages and increased fibrous tissue, creating a mass of reactive tissue known as *osteitis fibrosa cystica*. It is also known as *brown tumor*, the color of which can be attributed to the increased hemorrhage and hemosiderin deposition.

In recent years, osteitis fibrosa cystica has become very rare in hyperparathyroidism, owing to earlier detection of the disease. The common locations for the brown tumor are the ends of long bones, the pelvis, and ribs. Facial involvement is rare and, when present, usually involves the mandible [3].

In this article, we report a case of brown tumor of the mandible, maxilla, and patella as a first presentation of PHPT masked by severe vitamin D deficiency in a patient with a previous history of only repeated fractures. We discuss the patient’s unusual age at presentation; the uncommon location of the tumor; its symptomless late presentation; and the use of a less aggressive, conservative, multidisciplinary approach to the treatment of the tumor. This case also emphasizes the need to consider PHPT early in the differential diagnosis of tumorous lesions of the jaw. The patient was followed for 3 years postoperatively to assess the effectiveness of surgery alongside adjunctive treatment in maintaining low parathyroid hormone (PTH) levels and regression of the brown tumor lesions, as well as to assess the patient’s satisfaction in the long term.

## Case presentation

A 21-year-old, unmarried Arab woman with no known comorbidities presented with a massive swelling on the right side of her face that had been slowly increasing in size for the past 3 years, as well as with difficulty in bending her left knee. The swelling was large enough to cause trismus, and the patient was unable to open her right eye fully. She had no associated pain, bleeding, or superficial ulceration of the mass. The history taking for her presenting illness revealed multiple admissions for recurrent fractures. She had experienced no associated headaches, psychiatric manifestations, renal stones, or abdominal pain. She had no history of weight loss, fever, or night sweats. She had a negative family history for any tumors or disorders of calcium homeostasis. She also displayed psychological distress and concern regarding the impact of the mass on her appearance. Her social functioning and activities of daily living were markedly affected.

**Fig. 1:**
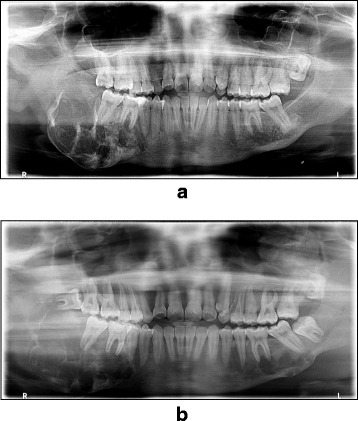
Panoramic x-ray of the patient’s teeth showing right mandibular swelling and an impacted left lower wisdom tooth. **a** Preoperative x-ray. **b** Postoperative intralesional steroid injection and removal of the left lower wisdom tooth

An extraoral examination showed a nontender hard swelling on the right side of the mandible that was causing facial deformity. An intraoral examination revealed a nontender firm swelling that was expanding the buccal mucosa and lingual mandibular plates, obliterating the buccal sulcus, and extending from the right premolars to the ascending ramus. The patient had a displaced, partially impacted tooth 18, mobile teeth 36 and 37, and nontender swelling of the left maxillary region with obliteration of the upper left buccal sulcus. A panoramic x-ray of the patient’s teeth showed a right mandibular swelling and an impacted left lower wisdom tooth (Fig. 1).

The patient’s thyroid examination result was normal. She had no associated lymphadenopathy or skeletal deformities. An examination of the lower extremities showed that the left knee had mild limitation of movement. The patient had no tenderness, erythema, or swelling of the knee joint or proximal leg on the left side. An examination of the patient’s right lower extremity was unremarkable.

She initially presented as an outpatient to the maxillofacial surgery department. The mass was suspected to be malignant, and a biopsy of the lesion was taken. The biopsy report showed a giant cell lesion and was referred to the endocrinology department to rule out metabolic bone disease. Her lesion was identified as a brown tumor of hyperparathyroidism, and laboratory investigations were done (Table 1).

**Table 1:** Time line of the patient’s serial laboratory investigations

Investigations	Normal values	Initial values	After 4 months of vitamin D therapy	Postsurgery	2-month follow-up	3-year follow-up
PTH, pg/ml	5–68.3	1652.7	564.6	154.6	222	103.3
Calcium, mmol/L	2.22–2.64	2.16	3.02	2.23	2.35	2.36
Phosphorous, mmol/L	0.70–1.50	0.71	0.94	0.81	1.22	0.93
Alkaline phosphatase, U/L	54–144	875	444	363	336	83
25(OH)D, ng/ml	40–100	5.84	18.3	15.2	29.5	24.8
Serum creatinine, U/L	10–70	41.0	35.0	52.0	51.0	42.0
Estimated glomerular filtration rate, ml/minute/1.73 m^2^	>90	144.28	150.93	132.5	133.35	139.21

*PTH* parathyroid hormone

The initial impression was of secondary hyperparathyroidism due to severe vitamin D deficiency, although the patient’s PTH level was remarkably high. The other suspicion was of PHPT masked by vitamin D deficiency.

**Fig. 2:**
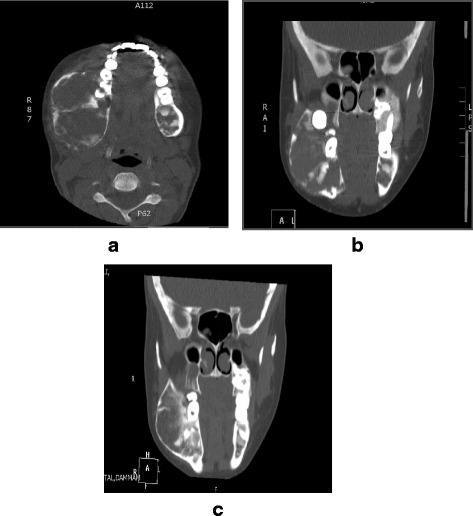
Maxillofacial computed tomographic scans with contrast enhancement showing multiple expansile lesions with ossification. The largest lesion can be seen within the body and ramus of the mandible. **a** Preoperative scan. **b** Scan obtained 5 months postoperatively demonstrating increased ossification of the lesion. **c** Scan obtained 1.5 years postoperatively showing reduction in the size of the lesion

Maxillofacial computed tomography (CT) with contrast enhancement showed multiple maxillofacial expansile lesions with ground-glass ossification. The largest lesion within the right mandible involved the body and ramus. It measured 6.4 × 4.1 cm. The largest lesion in the left maxilla involved the alveolar edge and measured 3.6 × 3.3 cm. No lymphadenopathy was seen (Fig. 2a).

**Fig. 3:**
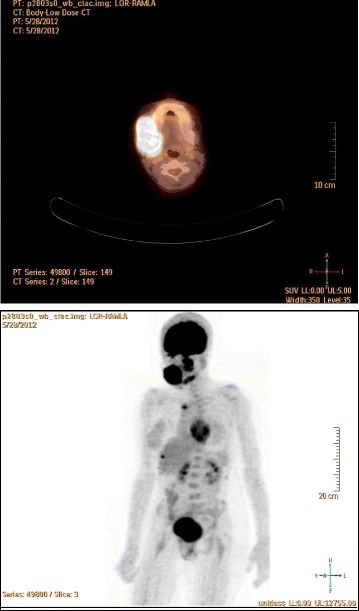
Positron emission tomography computed tomographic scan showing multiple hypermetabolic, expansile, bony lytic lesions in the right mandible, left maxillary sinus, left mandible, and manubrum sterni

A positron emission tomography CT scan showed multiple hypermetabolic, expansile, bony lytic lesions in the right mandible, left maxillary sinus, left mandible, and manubrum sterni (Fig. 3).

**Fig. 4:**
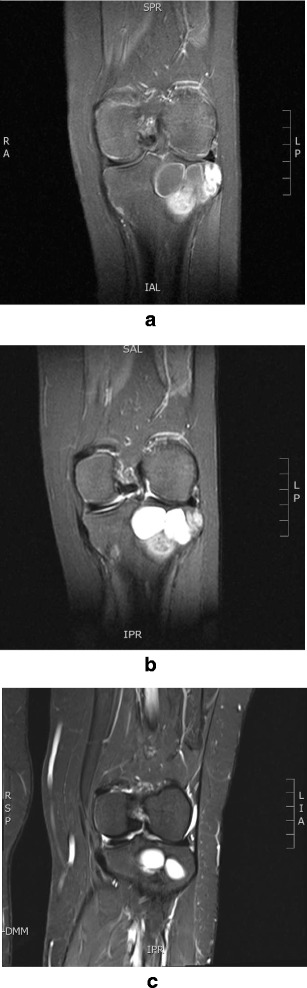
Magnetic resonance imagining of the patient’s left knee showing a heterogeneous, solid, and soft tissue mass involving the proximal epimetaphysis of the tibia and extending to the subarticular cortex. **a** Preoperative scan. **b** Scan obtained 6 months postoperatively showing a mild decrease in the size of the lesion with increased sclerosis of the solid component. **c** Scan obtained 2.5 years postoperatively showing a further decrease in the size of the mass

Magnetic resonance imaging (MRI) of the left lower extremity showed a heterogeneous, solid, and soft tissue mass involving the proximal epimetaphysis of the tibia and extending to the subarticular cortex. The mass was approximately 4.8 × 4.2 cm in size (Fig. 4a).

**Fig. 5:**
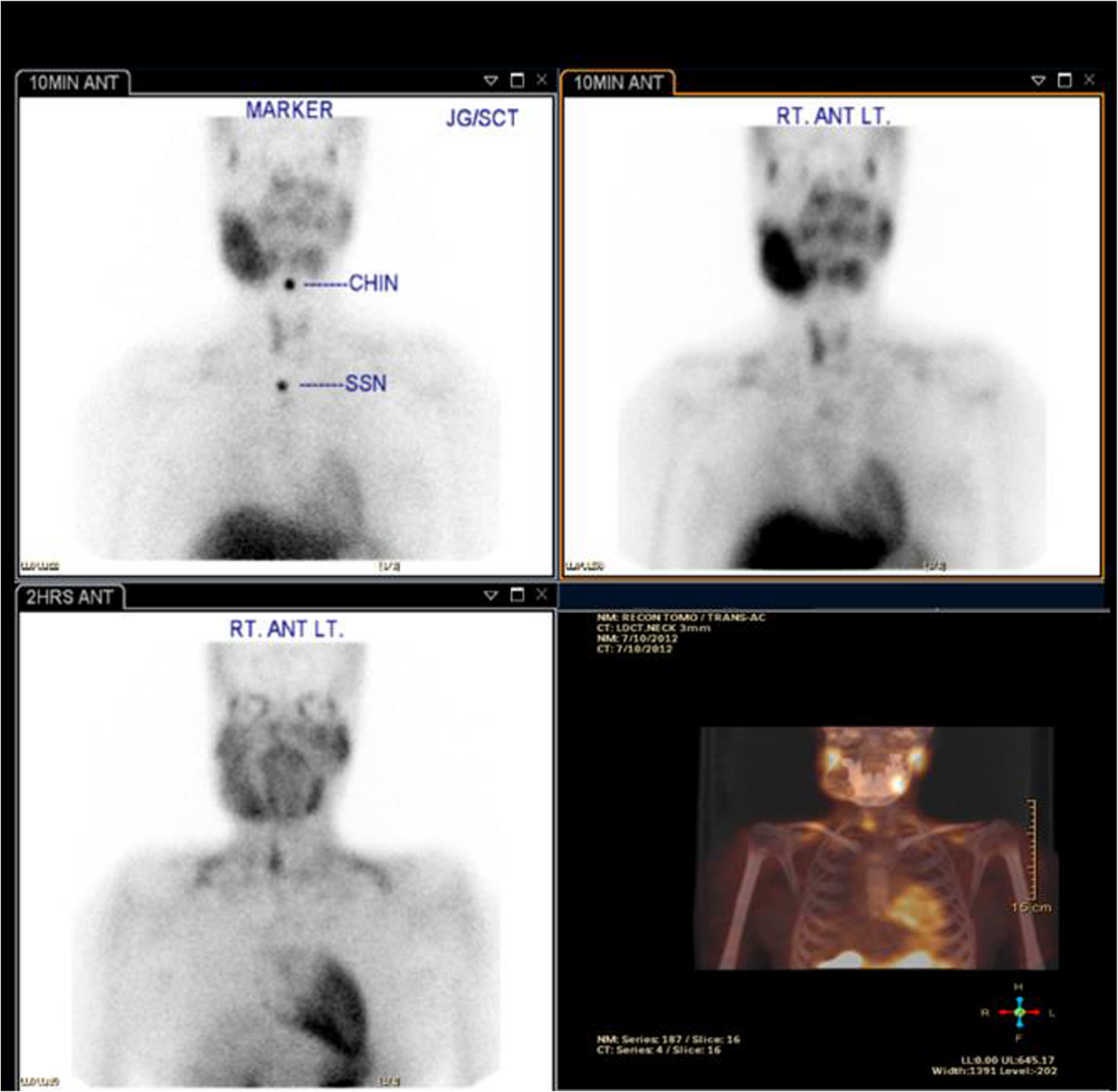
Technetium Tc-99 m sestamibi parathyroid study showing increased activity in the soft tissue mass, approximately 13.0 mm in size and lying behind the lower part of the right thyroid lobe

The patient was started on vitamin D 50,000 U by mouth weekly for 6 weeks. Her PTH levels decreased after 4 months on vitamin D therapy, but her calcium levels rose significantly (Table 1). The vitamin D dose was reduced because of fear of hypercalcemia, and the patient was kept on 5000 U weekly thereafter. An ultrasound scan of the parathyroid gland was done at the time, which showed a right lower lobe parathyroid solid nodule. A technetium Tc-99 m sestamibi parathyroid study showed increased activity in the soft tissue mass, approximately 13.0 mm in size and lying behind the lower part of right thyroid lobe, suggestive of parathyroid adenoma (Fig. 5).

She was referred to surgical oncology and underwent right lower parathyroid adenoma excision. Her PTH levels improved significantly after surgery (Table 1). Postoperatively, she had a brief episode of hypocalcemia (1.97 mmol/L), and she was started on calcium carbonate 1200-mg tablets twice daily for 2 weeks. She was received maxillofacial follow-up for intralesional steroid 60-mg methylprednisolone depot injection as well as endocrinological follow-up for vitamin D therapy. She was followed with frequent laboratory and radiological investigations for the next 3 years, through the reporting of this case, and the masses have shrunk in size.

Maxillofacial contrast-enhanced CT performed 5 months after resection of the parathyroid adenoma showed lesions with more ossification, which could be a sign of healing. The largest right mandibular lesion was stable in size, but the left maxillary mass was reduced in size to 3 × 2.5 cm (compared with 3.6 × 3.3 cm previously) (Fig. 2b). CT performed 1.5 years postsurgery showed reduction in size of the right mandibular lesion to 5.5 × 4.0 cm (compared with 6.4 × 4.1 cm previously), and the maxillary lesion was further reduced to 2.3 × 2.2 cm (Fig. 2c).

MRI of the left knee with contrast enhancement performed 6 months postsurgery showed a mild decrease in size since the previous study, with increased sclerosis of the solid component (Fig. 4b). MRI of the left knee performed 2.5 years postsurgery showed further reduction in the size of the lesion from the previous study (Fig. 4c).

Overall, the patient showed serological, radiological, clinical, and psychological improvement from her initial condition. Although we were not able to retrieve an initial photograph for comparison, we took a picture of the mass after its marked reduction 1.5 years postoperatively.

## Discussion

Brown tumors, albeit more commonly found in PHPT, can also be seen with secondary causes such as vitamin D deficiency or renal disease. Our patient demonstrated overlap between PHPT caused by parathyroid adenoma and vitamin D deficiency secondary to hyperparathyroidism. Before the 1970s, PHPT was a disease characterized by recurrent kidney stones, brown tumors, neuromuscular dysfunction, and symptomatic hypercalcemia [4]. Today, it can be diagnosed in the early and asymptomatic period because of advances in blood analysis and a growing awareness of this disease [5]. However, the case of our patient highlights the significance of keeping brown tumors high on the list in the differential diagnosis of a patient presenting with a bony mass, even if the patient shows no signs of hyperparathyroidism and the patient’s calcium levels are normal or subnormal.

The name *tumor* is a misnomer because the lesion, although invasive in some instances, does not have a neoplastic potential and should be differentiated from true giant cell tumors of bone [6]. Brown tumors are very similar to giant cell tumors, but in the context of hyperparathyroidism they are considered reparative granulomas [7].

When a patient—especially a middle-aged patient—presents with unexplained lytic bone lesions or pathological fractures, surgeons, endocrinologists, and especially radiologists should be reminded of this unusual presentation of PHPT to avoid unnecessary surgical removal [8]. Radiographic findings can mimic bone malignancy, while the synchronous involvement of multiple skeletal segments can be interpreted as diffuse metastatic disease [9].

There are very few case reports in the literature describing Brown tumor localized in the maxillary and facial regions in PHPT [10]. In the case of a lesion in the lytic region of the jaw bones, the most likely diagnoses would include hyperparathyroidism-jaw tumor syndrome, odontogenic cysts and tumors (radicular cyst, lateral periodontal cyst, and ameloblastoma), infectious diseases (bone abscess, localized osteomyelitis), metabolic bone disease hyperparathyroidism, metastasis from a known or an unknown primary site (lung, breast, kidney, prostate), and primary bone tumors and cysts (simple bone cyst, eosinophilic granuloma, giant cell lesions, odontogenic keratocyst, myxoma, and odontogenic fibroma) [7].

Total or subtotal parathyroidectomy is the gold standard for the treatment of PHPT [11], which we performed in our patient. The following is a summary of the National Institutes of Health guidelines [12] for parathyroidectomy in asymptomatic patients:

1. Serum calcium at least 1 mg/dl above the upper limit of normal
2. Creatinine clearance reduced by more than 30 % as compared with age-matched controls
3. T-score less than or equal to −2.5 or fragility fracture
4. Age younger than 50 years

As reviewed in Table 1, although postoperatively our patient’s PTH levels reached normal limits, it is important to closely follow these patients for recurrence and fluctuations in hormone levels. In addition, as an adjunct to surgery, we used pharmacotherapy to maintain our patient’s serum hormone levels and reduce her brown tumor further to negate the need for further surgical procedures. She is being maintained on vitamin D_3_ 5000 U/week, along with intralesional steroids.

We reviewed three studies published in high-impact journals in which authors advocated the use of high vitamin D doses safely in patients with PHPT. In one study done in 2014, authors stated that vitamin D replacement in mild PHPT with coexistent vitamin D deficiency reduces parathyroid levels significantly without signs of hypercalcemia or hypercalciuria [13, 14]. Another retrospective study demonstrated that 25(OH)D increased significantly (*P* < 0.0001) from a baseline of 14.65 ± 6.57 ng/ml to 42.17 ± 12.98 ng/ml after weekly treatment with 50,000 IU of vitamin D, but that pre- and posttreatment unadjusted serum calcium remained stable in the high-dose group [15].

We administered a 50,000-IU weekly dose of vitamin D in our patient, and at her 4-month follow-up her serum calcium levels had risen to 3.02 mmol/L. After her 4-month follow-up, we reduced the dose to 5000 IU weekly and her calcium levels returned to baseline. We suggest that further trials be done to study the effect of high-dose vitamin D in different patient populations, and inclusion and exclusion criteria should be developed to assess the candidates in whom high doses can safely be administered without increasing the risk of hypercalcemia and/or hypercalciuria and renal stones.

The overall outcome of our less aggressive approach relieved the patient of unnecessary anxiety regarding an additional surgical intervention and its consequences. Our patient was pleased with the cosmetic outcome and the progress of her recovery.

The limitations in the management of this case were due to lack of specific guidelines for management of vitamin D deficiency in patients with features of both PHPT and secondary vitamin D deficiency-induced hyperparathyroidism. In addition, we were unable to retrieve a photograph of the patient from the initial presentation for comparison in this case report. However, the main strength of our management was in early referral and involvement of multiple specialists in the appropriate care of our patient. This multidisciplinary approach prevented us from misdiagnosis of the tumor and from performing deforming radical surgeries in the maxillofacial region. Working in a specialist center and having a cooperative patient also allowed us to carry out all necessary diagnostic and therapeutic interventions without any delay. We were able to follow our patient very closely with blood investigations of vitamin D, parathyroid hormone, calcium, and phosphorous levels to determine the causation between high-dose vitamin D therapy and hypercalcemia.

## Conclusions

On the basis of this case report, we stress the significance of actively involving radiologists, endocrinologists, surgical oncologists, and maxillofacial surgeons in the management of this type of case. In addition, it should be kept in mind that atypical age demographics and tumor sites should not rule out brown tumor as a differential diagnosis. Last, the patient’s laboratory and radiological investigations should always be supported by clinical impression, and the psychological impact of the disease on the patient should be one of the deciding factors for establishing the management plan, as it was for us.
